# Retraction of: Scottish Firefighters Occupational Cancer and Disease Mortality Rates: 2000-2020

**DOI:** 10.1093/occmed/kqaf087

**Published:** 2025-10-01

This is a retraction of: A. A. Stec, A. Robinson, T. A. M. Wolffe, E. Bagkeris, Scottish Firefighters Occupational Cancer and Disease Mortality Rates: 2000–2020, Occupational Medicine, Volume 73, Issue 1, January 2023, Pages 42–48, https://doi.org/10.1093/occmed/kqac138.

Following publication, it was brought to the Editors’ attention that while the authors compared their data with similar studies on cancer as an occupational risk in firefighters, the methodologies differed, particularly with respect to Standardized Mortality Ratios (SMRs).

The journal published an Expression of Concern^1^ and commissioned an independent scientific review. The Editors asked the authors to amend their paper in relation to Relative Risks, and their conclusions based upon them, outlining the differences and their reasons for the chosen approach. The authors proposed new text clarifying their approach which did not change their conclusions. As the two parties could not agree, the Editors are retracting this article because they have concerns regarding the published findings in the absence of further explanation of methodological differences. The authors disagree with this assessment and their paper’s retraction.

^1^Expression of Concern: Scottish Firefighters Occupational Cancer and Disease Mortality Rates: 2000-2020. Occupational Medicine, Volume 75, Issue 3-4, April 2025, Page 215, https://doi.org/10.1093/occmed/kqaf032

